# Eudragit S100 Coated Citrus Pectin Nanoparticles for Colon Targeting of 5-Fluorouracil

**DOI:** 10.3390/ma8030832

**Published:** 2015-02-27

**Authors:** M. Biswaranjan Subudhi, Ankit Jain, Ashish Jain, Pooja Hurkat, Satish Shilpi, Arvind Gulbake, Sanjay K. Jain

**Affiliations:** Department of Pharmaceutical Sciences, Dr. Hari Singh Gour Central University, Sagar 470 003, (MP), India; E-Mails: bisu_ai75@yahoo.co.in (M.B.S.); ankitjainsagar@gmail.com (A.J.); ashish.g.jain@gmail.com (A.J.); pooja159@gmail.com (P.H.); shilpisatish@gmail.com (S.S.); arvind.gulbake@gmail.com (A.G.)

**Keywords:** Eudragit S100, citrus pectin, 5-Fluorouracil, colon targeting, cancer

## Abstract

In the present study, Eudragit S100 coated Citrus Pectin Nanoparticles (E-CPNs) were prepared for the colon targeting of 5-Fluorouracil (5-FU). Citrus pectin also acts as a ligand for galectin-3 receptors that are over expressed on colorectal cancer cells. Nanoparticles (CPNs and E-CPNs) were characterized for various physical parameters such as particle size, size distribution, and shape *etc. In vitro* drug release studies revealed selective drug release in the colonic region in the case of E-CPNs of more than 70% after 24 h. *In vitro* cytoxicity assay (Sulphorhodamine B assay) was performed against HT-29 cancer cells and exhibited 1.5 fold greater cytotoxicity potential of nanoparticles compared to 5-FU solution. *In vivo* data clearly depicted that Eudragit S100 successfully guarded nanoparticles to reach the colonic region wherein nanoparticles were taken up and showed drug release for an extended period of time. Therefore, a multifaceted strategy is introduced here in terms of receptor mediated uptake and pH-dependent release using E-CPNs for effective chemotherapy of colorectal cancer with uncompromised safety and efficacy.

## 1. Introduction

Colorectal cancer is the second leading cause of cancer-related mortality with about 655,000 deaths worldwide every year. Conventional therapies for colorectal cancer include surgery, chemotherapy, and radiation therapy. Conventional chemotherapy is not very effective as the drug does not reach the target site in effective concentrations. Thus, effective treatment demands an increased dose size, which may lead to undue side effects [1]. To overcome these situations, a colon specific drug delivery approach is employed to bring about favorable consequences *i.e.*, minimized drug distribution to non-target cells in order to shun untoward effects, improved targeting ability to cancer cells, and controlled release (temporal effects) of cytotoxic drug to cancer cells in effective concentration. As a drug of choice, 5-fluorouracil (5-FU) is selected because of designated first line therapy for colorectal cancer [2].

In the past, a number of polymeric materials were used for the preparation of nanoparticles. Recently, polysaccharides have been investigated for the preparation of nanoparticles because of their excellent physicochemical properties and biocompatible nature which are beneficial for biomedical use [3,4,5]. Pectins are a family of complex polysaccharides that contain large amounts of poly-(d-galactouronic acid) bonded via α-1,4-glycosidic linkage. They are heterogeneous moieties with respect to chemical structure and molecular weight [6] and can be classified into low methoxy (LM), high methoxy (HM) and amidated pectins. Pectins have a number of pharmaceutical applications and are presently considered as promising biodegradable carriers for colon-specific drug delivery for systemic action or topical treatment of diseases such as ulcerative colitis, Crohn’s disease, and colon carcinomas, as indicated by a plethora of studies published over the last decennia [7,8,9,10]. Various techniques have been reported to manufacture the pectin-based drug delivery systems, especially ionotropic gelation and gel coating [11,12]. The most striking property of pectins for industrial applications is their gelling activity. Factors governing gelation and thereby influencing gel characteristics are the type and concentration of pectin, the modifications of hydroxyl group, the pH of pectin solution, the temperature and the presence of cations [13]. HM-pectin forms gels with sugar and acid. HM-pectin, unlike LM-pectin, does not contain sufficient acid groups to gel or precipitate with calcium ions. The mechanism of LM-pectin gelation relies mainly on the well-known “egg-box” model [14]. Amidation further improves the gelling potential of LM-pectin. Amidated pectins need less calcium to gel and are less prone to precipitation at high calcium levels [15]. When solution pH is raised, the polycarboxylate groups are ionized, and able to react with calcium ions to form calcium-pectinate gels. The interaction of calcium ions and the carboxylate groups of pectin results in intermolecular chelation leading to the formation of macromolecular aggregates [16]. This property of pectin was employed to form the nanoparticulate system. Various drug delivery approaches have been developed for colon-specific drug delivery, which include a pH-sensitive system, a time-dependent system, pro-drugs, and a microflora-activated system to deliver therapeutic agents to the desired colonic sites; hence systemic drug absorption should be reduced as this leads to unwanted systemic side effects [17,18].

Of all the systems formulated for colon-specific drug delivery, the pH-sensitive system and time-dependent system are mostly used [8,9,10]. The pH variation along the GI tract (GIT) is based on the strategy of using pH as a trigger to achieve drug release in the colon. The high individual variability together with similarity in pH between the small intestine and the colon make the site specificity of the pH-dependent system not very reliable [11,12].

Eudragit S 100 is an anionic copolymer based on methacrylic acid and methyl methacrylate. The ratio of the free carboxyl groups to ester groups is approx. 1:2. It was selected for coating of citrus pectin nanoparticles due to its desirable attributes such as pH responsive nature *i.e.*, no release of entrapped drug at low pH of the stomach as well as in the small intestine where the pH is almost neutral. In this way, it imparts a colon specific nature to the delivery system.

Thus, in the present project, citrus pectin and Eudragit S100 (pH-responsive enteric polymer) were chosen to fabricate nanoparticulate drug delivery systems for site-specific delivery of 5-FU for the effective treatment of colorectal cancer. The proposed delivery system is premised to protect the drug loss in the upper GI tract because of the deterrent nature of Eudragit S100 (ES) to the milieu of the upper GI tract and to deliver 5-FU upon reaching the colon due to the amiable pH of the colonic fluid. Moreover, citrus pectin being biodegradable in nature also acts as a ligand for the galectin-3 receptors which are over expressed on the colorectal cancer cells [19]. Thus, a multifaceted approach is conceived which includes biodegradability and colon selectivity in terms of receptor mediated uptake and pH-dependent release of 5-FU using Eudragit S100 coated citrus pectin nanoparticles (E-CPNs) for a better treatment of colorectal cancer.

## 2. Materials and Methods

The 5-FU was received as a gift sample from Biochem (Mumbai, India). Eudragit S100 was procured as gift sample from Evonik Degussa India Pvt Ltd (Mumbai, India). Pectin (P9135, pectin from citrus peel, galacturonic acid g ≥74.0%) was purchased from Sigma-Aldrich (Mumbai, India). Pancreatin (from pig pancreas), pepsin (bovine) and dialysis membrane (Molecular weight cut-off 3.5 kD) were procured from Himedia (Mumbai, India). Pectinase was purchased from Loba Chemie (Mumbai, India). All other chemicals used were of analytical grade. Double distilled water (DDW) was used throughout the study wherever needed.

### 2.1. Preparation of Citrus Pectin Nanoparticles (CPNs)

Citrus pectin nanoparticles (CPNs) were prepared using the method reported by Yu *et al.* (2009) with suitable modifications [20]. Briefly, citrus pectin (100 mg) was added to 5 mL of DDW under constant mechanical stirring (Remi, Mumbai, India) for 1 h at 1000 rpm and then immersed in a water bath at 55 °C for 30 min to obtain a solution with a viscosity of 80 cps, as measured by a rotational viscometer (Brookfield Digital DV-E Viscometer, Middleboro, MA, USA). Then, powdered 5-FU (75 mg) was added to the system maintained at 55 °C with stirring (1000 rpm for 1 h). After that, 1 mL of calcium hydroxide solution (0.025 M) was added to the system drop-wise with continuous stirring for an additional 1 h to obtain a mixture with a viscosity of 90 cps. The system was kept stirring at 55 °C for 2 h and then cooled rapidly in a water bath to 20 °C. After 30 min, the mixture was put into a dialysis bag and dialyzed against 500 mL of DDW at 20 °C for 24 h to obtain the drug-loaded nanoparticles (CPNs). These CPNs were then lyophilized (Freeze-dryer, Labconco, Kansas City, MI, USA) to obtain a dry form.

### 2.2. Coating of CPNs

The coating of CPNs was performed by a simple solvent evaporation method as reported by Maestrelli *et al.* (2008) [21]. The enteric coating solution was composed of Eudragit S100 in acetone (12% w/v). Coating was obtained by dispersing 100 mg of CPNs in coating solution with a core: coat ratio of 1:10 followed by solvent evaporation in a rotary evaporator (Super Fit, Ambala, India). The process was repeated until the desired amount of coating was achieved. Samples of coated nanoparticles (E-CPNs) were then dried and weighed (Table S1).

### 2.3. Characterization of CPNs and E-CPNs

#### 2.3.1. Particle Size

The average particle size and polydispersity index of the nanoparticles (CPNs and E-CPNs) were determined by photon correlation spectroscopy using a Zetasizer DTS ver. 5.03 (Malvern Instrument, Worcestershire, England). The samples of nanoparticles dispersions were diluted to four times their volume with 0.05 M NaCl. The particle size and PDI are represented by the average (diameter) of the Gaussian distribution function in the logarithmic axis mode.

#### 2.3.2. Zeta Potential

The zeta potential of nanoparticles (CPNs and E-CPNs) was determined by measurement of the electrophoretic mobility applying the Helmholtz-Smoluchowsky equation. For measurement of zeta potential, Zetasizer DTS ver. 5.03 (Malvern Instrument, Worcestershire, UK) was used. Samples were adjusted to a conductivity of 80 μS/cm with a solution of 0.05M NaCl at field strength of 20 V/cm.

#### 2.3.3. Particle Shape and Surface Morphology

##### Transmission Electron Microscopy

**Figure 1:**
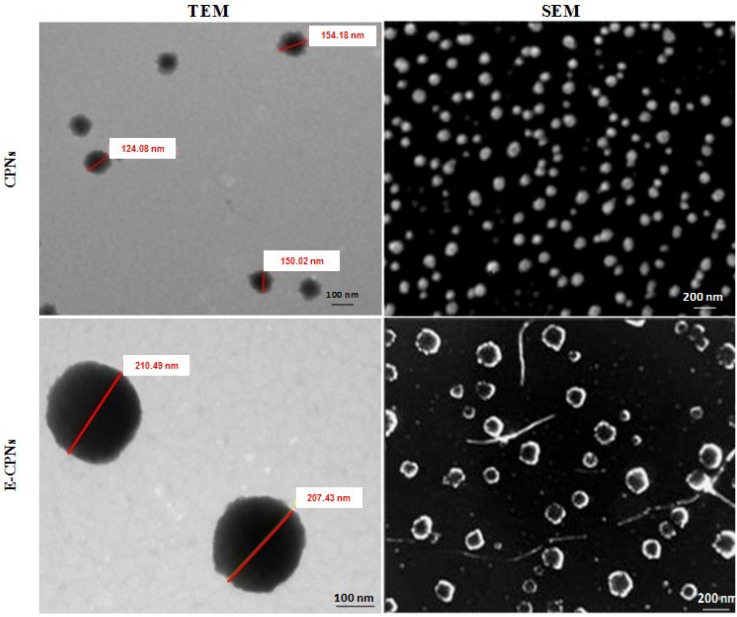
Transmission electron microscopy (TEM) and scanning electron microscopy (SEM) images of nanoparticles, *i.e.*, Citrus pectin nanoparticles (CPNs) and Eudragit S100 coated citrus pectin nanoparticles (E-CPNs).

The nanoparticles (CPNs and E-CPNs) were characterized for their shape by transmission electron microscopy (Philips Morgagni 268D, Eindhoven, Netherlands). The samples (10 µL) were adhered to coated grids and negatively stained with 2% (w/v) phosphotungstic acid for 90 s, and then blot dried using filter paper. The grid was allowed to dry carefully in air, and samples were examined under transmission electron microscope at suitable magnification (Figure 1).

##### Scanning Electron Microscopy

The nanoparticles (CPNs and E-CPNs) were characterized for their morphology by scanning electron microscopy (LEO 435 VP, Eindhoven, The Netherlands). To prepare the samples, a double-adhesive tape was stuck to an aluminum stub and removed to obtain an adhesive coated aluminum stub. Then, 1–2 drops of nanoparticles dispersion were applied on the stub and dried overnight in a desiccator. The stubs were coated with gold to a thickness of ~300 Å under an argon atmosphere using a gold sputter coater in a high-vacuum evaporator. The coater was operated at 0.1 torr (argon) for 90 s at an accelerating voltage of 15 kV. The coated samples were then randomly investigated using a scanning electron microscope at suitable magnification (Figure 1).

#### 2.3.4. Entrapment Efficiency

The nanoparticles (CPNs and E-CPNs) weighing 50 mg were digested in 5 mL of pectinase solution (120 FDU/mL, pH 7.0) for 12 h [22]. The digested homogenate was centrifuged (Remi, Mumbai, India) at 3000 rpm for 5 min, and the supernatant was analyzed for 5-FU content using a double beam UV spectrophotometer at λ_max_ 266.0 nm. The percent entrapment efficiency (%EE) and percent drug loading (%DL) were calculated using the formulae (Table 1):

(1) $$\text{\% EE }=\frac{\text{Mass of drug in nanoparticles}}{\text{Mass of drug used in formulation}}\times 100$$

(2) $$\text{\% drug loading}(\text{DL})=\frac{\text{Mass of drug in nanoparticles}}{\text{Mass of nanoparticles recovered}}\times 100$$

**Table 1:** Characterization of nanoparticles.

Formulation code	Particle size (nm)	PDI	Zeta potential (mV)	% Entrapment efficiency	% Drug loading
CPNs	174.65 ± 5.32	0.095	−18.4 ± 0.4	38.75 ± 0.74	21.45 ± 0.87
E-CPNs	218.12 ± 10.25	0.117	−27.5 ± 0.8	35.15 ± 0.52	20.84 ± 0.75

Values represent Mean ± SD, *n* = 3.

### 2.4. In vitro Release Study

#### 2.4.1. In Simulated Gastric Fluids of Different pH Conditions

*In vitro* drug release studies were carried out according to the Souder and Ellenbogen extraction technique with slight modifications [23]. The intention of using the simulated fluids of different pH was to mimic mouth-to-colon transit in the following manner: (a) 1–2 h: simulated gastric fluids (SGF) of pH 1.2; (b) 3–4 h: mixture of simulated gastric and intestinal fluids of pH 4.5; (c) 5–6 h: simulated intestinal fluids (SIF) of pH 6.8; (d) 7–24 h: simulated colonic fluid (SCF) pH 7.0.

**Figure 2:**
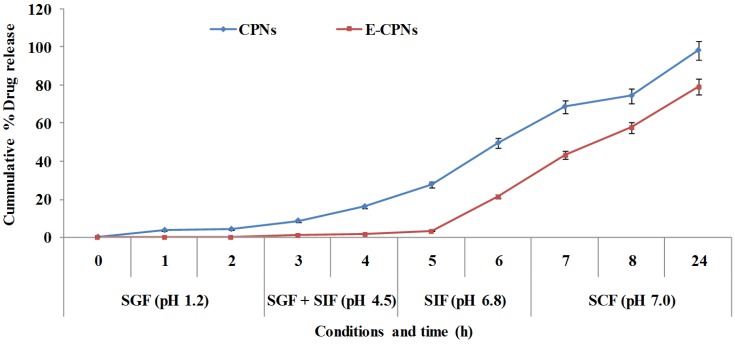
*In vitro* drug release profiles of CPNs & E-CPNs in different simulated GI tract (GIT) fluids over a period of time (Mean ± SD, *n* = 3). [GIT: Gastro-intestinal tract; SGF: Simulated gastric fluid; SIF: Simulated intestinal fluid; SCF: Simulated colonic fluid].

*In vitro* drug release patterns from nanoparticles (CPNs and E-CPNs) were studied using a dialysis bag [24]. Nanoparticles (50 mg) taken in a dialysis bag (MWCO 3.5 kD) were placed in a beaker containing 100 mL of simulated fluids at 37 ± 1 °C with slow magnetic stirring under perfect sink conditions and the fluids were changed as per the Souder and Ellenbogen scheme at a definite time point. Samples (1 mL) were withdrawn periodically and replaced with the same volume of fresh dissolution medium. The amount of drug released was determined using UV-spectroscopy at λ_max_ 266.0 nm and reported as the percent cumulative drug release in Figure 2.

#### 2.4.2. *In vitro* Drug Release Study in the Presence and Absence of Rat Caecal Content

To overcome the limitations of conventional dissolution tests for evaluating the performance of colon specific drug delivery systems triggered by colon specific bacterial flora, rat caecal content has been utilized as an alternative dissolution medium so called rat caecal content medium or simulated colonic fluid. This medium was prepared using the method as reported by Van den Mooter *et al.* (1994) [25]. Rats weighing 120–150 g were taken and maintained on normal diet. Citrus pectin solution (1 mL, 2% w/v) was administered through the oral route and this treatment was continued for seven days in order to induce the enzymes specific for the biodegradation of the pectin during its passage through the colon. Rats were sacrificed by spinal traction for 45 min prior to commencement of drug release studies. The abdomen was opened, the caecum was traced, ligated at both ends, dissected, and immediately transferred into PBS (pH 7.0) bubbled previously with CO_2_. The caecal bags were opened and their contents were individually weighed, pooled, and suspended in the buffer continuously bubbled with CO_2_. They were finally added to the dissolution media to give a final caecal dilution of 2% w/v. The suspension was then filtered through cotton wool and sonicated using a probe sonicator (Lark innovative technology, Chennai, India) at 950 W for 5 min at 4 °C to disrupt the bacterial cells. After sonication, the mixture was centrifuged at 2000 rpm for 20 min. As the environment in caecum is naturally anaerobic, all the operations were carried out under CO_2_ atmosphere [26].

**Figure 3:**
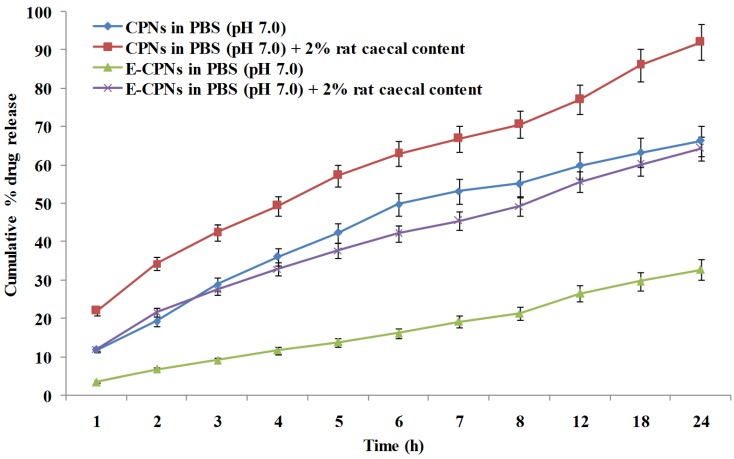
Cumulative % drug release profiles of CPNs and E-CPNs at various conditions (Mean ± SD, *n* = 3).

Drug release studies of nanoparticles (CPNs and E-CPNs) in the presence and absence of rat caecal content were carried out in sealed glass vials with 10 mL of dissolution medium maintained at 37 ± 2 °C under slow magnetic stirring. 50 mg of nanoparticles was placed in the dissolution medium (PBS, pH 7.0) containing 2% rat caecal contents. Samples (0.2 mL) were withdrawn after a fixed time interval of 1, 2, 3…. 8 and 24 h, volumes were made up to 10 mL and the same volume of dissolution medium was replaced with PBS (pH 7.0). The withdrawn samples were centrifuged at 2000 rpm for 10 min and the supernatant was filtered through Whatmann filter paper followed by estimation of 5-FU spectrophotometrically (Figure 3).

### 2.5. In vitro Cytotoxicity Studies

*In vitro* cytotoxicity potentials of free 5-FU solution and nanoparticles (CPNs and E-CPNs) suspension against HT-29 cells were compared using sulphorhodamine B assay (SRB assay) [27,28]. The cell lines were grown in RPMI 1640 medium containing 10% fetal bovine serum and 2 mM l-glutamine. For the present screening experiment, cells were inoculated into 96 well microtiter plates in 100 µL at plating densities, depending on the doubling time of individual cell lines. After cell inoculation, the microtiter plates were incubated at 37 °C, 5% CO_2_, 95% air, and 100% relative humidity for 24 h prior to addition of the formulation.

After 24 h, one 96 well plate containing 5 × 10^3^ cells/well was fixed *in situ* with trichloroacetic acid to represent a measurement of the cell population at the time of addition of the drug (Tz). Experimental samples (free drug and nanoparticles) were initially solubilized in dimethylsulfoxide at 100 mg/mL and diluted to 1 mg/mL using water and frozen prior to use. At the time of drug addition, an aliquot of frozen concentrate (1 mg/mL) was thawed and diluted to 100 μg/mL, 200 μg/mL, 400 μg/mL and 800 μg/mL with complete medium containing test article. Aliquots of 10 µL of these different drug dilutions were added to the appropriate microtiter wells already containing 90 µL of medium, resulting in the required final drug concentrations *i.e.* 10 μg/mL, 20 μg/mL, 40 μg/mL and 80 μg/mL.

#### Endpoint Measurement

After addition of different formulations, plates were incubated at standard conditions for 48 h and assay was terminated by the addition of cold TCA. Cells were fixed *in situ* by the gentle addition of 50 µL of cold 30% (w/v) TCA (final concentration, 10% TCA) and incubated for 60 min at 4 °C. The supernatant was discarded; the plates were washed five times with DDW and air dried. Sulforhodamine B (SRB) solution (50 µL) at 0.4% (w/v) in 1% acetic acid was added to each of the wells, and plates were incubated for 20 min at room temperature. After staining, unbound dye was recovered and the residual dye was removed by washing five times with 1% acetic acid. The plates were air dried. Bound stain was subsequently eluted with 10 mM trizma base, and the absorbance was read on a plate reader at a wavelength of 540 nm with 690 nm reference wavelength [29].

**Figure 4:**
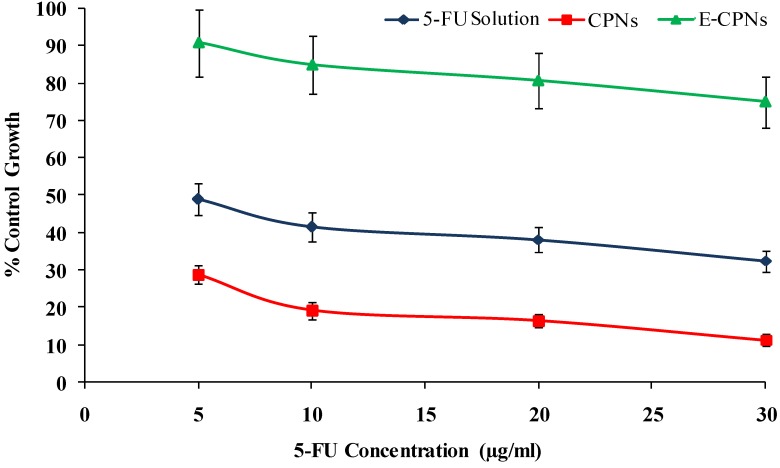
Cytotoxicity of 5-FU solution, CPNs and E-CPNs after 48 h incubation against HT-29 colorectal cancer cell lines (Mean ± SD, *n* = 3).

Percent growth was calculated on a plate-by-plate basis for test wells relative to control wells. Percent growth was expressed as the ratio of the average absorbance of the test well to the average absorbance of the control wells × 100. Using the six absorbance measurements [time zero (Tz), control growth (C), and test growth in the presence of drug at the four concentration levels (Ti)], the percentage growth was calculated at each of the drug concentration levels (Figure 4). Percentage growth inhibition was calculated as: [(Ti–Tz)/(C–Tz)] × 100 for concentrations having Ti ≥ Tz(Ti–Tz) positive or zero, and [(Ti–Tz)/Tz] × 100 for concentrations having Ti < Tz (Ti–Tz) negative. The dose response parameters were calculated for each formulation. Growth inhibition of 50% (GI_50_) is the drug concentration resulting in a 50% reduction in the net protein increase in control cells during the drug incubation and it was calculated from [(Ti–Tz)/(C–Tz)] × 100 = 50. The drug concentration resulting in total growth inhibition (TGI) was calculated from Ti = Tz. The LC_50_ (concentration of drug resulting in a 50% reduction in the measured protein at the end of the drug treatment as compared to initial) indicating a net loss of cells following treatment was calculated from [(Ti–Tz)/Tz] × 100 = −50.

### 2.6. In vivo Studies

#### 2.6.1. Experimental Protocol Approval

Albino rats (Wistar strain) of either sex weighing about 120–150 grams were used for the present *in vivo* study. The experimental protocol was duly approved by the Institutional Animal Ethics Committee, Dr. H. S. Gour Vishwavidyalaya, Sagar (M.P.), India. The formulations were administered orally in equivalent doses to rats and a comparison for their organ distribution profiles was made. These animals were kept in well-spaced ventilated cages and maintained on healthy and fixed diets (Bengal grams soaked in water). They were fasted overnight before the experiment but allowed access to water *ad libitum*.

#### 2.6.2. Blood Profile and GI Organ Distribution Study

**Figure 5:**
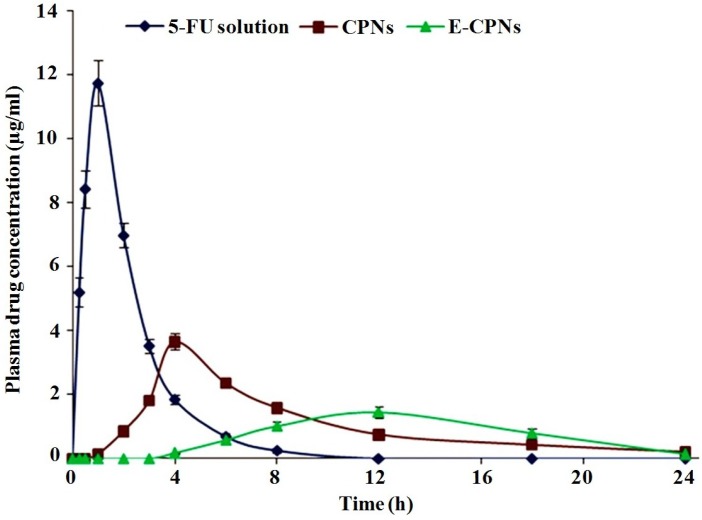
Mean plasma 5-FU concentration *vs.* time profile in albino rats after oral administration of various formulations (dose ~ 7.14 mg/kg) (Mean ± SD, *n* = 3).

**Figure 6:**
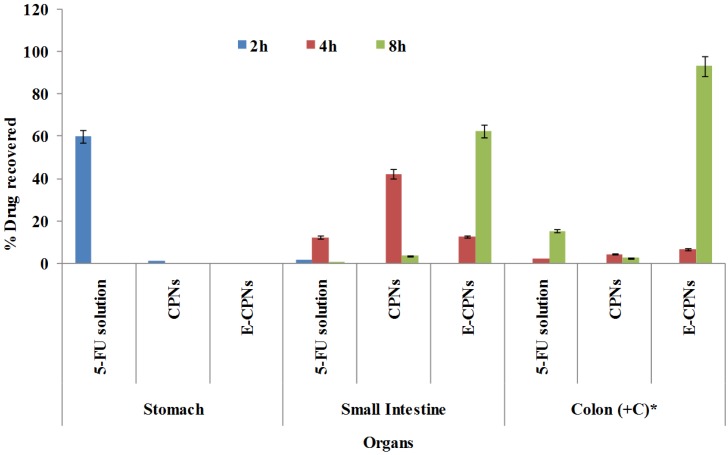
Distribution profiles of various formulations in different parts of GIT as a function of time (Mean ± SD, *n* = 3).

A total of 36 animals were used for this study (*n* = 3). The animals were divided into four groups. Each group contained three animals. All animals were administered orally with the help of cannula, the formulation contained drug solution equivalent to 7.14 mg/kg body weight of the animal. Group 1 received no drug and acted as control. Group 2 received 5-FU solution while Group 3 and Group 4 received CPNs and E-CPNs, respectively. After the administration of the drug, one animal from each group was sacrificed at each time interval *i.e.*, 2 h, 4 h, and 8 h. Before sacrificing the animals, a blood sample (0.1 mL) was taken from the retro-orbital plexus using a uniformly tapered capillary at stated time points. Samples were collected in eppendorfs over 20 µL heparin sodium (22 mg/mL) and centrifuged (3000g, 10 min) at 4 °C to separate plasma [30]. Cold acetonitrile was added to these samples and centrifuged (5000g, 10 min) at 4 °C. The supernatants were collected and filtered through 0.22 µm syringe filters and concentrations were determined by an HPLC method [31]. The mobile phase consisted of methanol: water (10:90, v/v) adjusted to pH 3.2 with perchloric acid at a flow rate of 1.0 mL/min. The HPLC column was a C18 (4.6 mm× 250 mm, 5 µm) and the results are shown in Figure 5. For GI organ distribution studies, GI tract (GIT) was removed and the mesenteric and fatty acid tissues were separated. The GIT was segmented into the stomach, small intestine, caecum and colon. The luminal contents were removed by applying gentle pressure with wet scissors to the tissues. Organs and luminal contents were weighed. The GIT parts were cut open longitudinally and rinsed with PBS (pH 7.4) to remove any remaining luminal contents. The entire tissue part of each organ was homogenized with PBS (pH 7.4) using tissue homogenizer (MAC Micro Tissue Homogenizer, New Delhi, India) at 4 °C. Either 1g of each organ or the whole GIT part/tissue was used in the case if the GIT part was found to be less than 1g. To the tissue homogenates, an equal volume of acetonitrile was added and mixed with vortex (30 s). Then, the tissue homogenates were centrifuged (2000g, 10 min). The fatty layer was discarded and supernatants were collected. Luminal contents were diluted with PBS (pH 7.4) followed by centrifugation, and supernatants were collected. Supernatants from all the above samples were filtered through a 0.45 μm membrane filter, and analyzed for 5-FU using the HPLC method [31]. The amount of drug recovered in different segments of the GI tract at different time intervals was calculated and is presented in Figure 6.

## 3. Statistical Analysis

All results were expressed as mean ± standard deviation (SD) and statistical analysis was performed with NCSS 2007 Version 07.1.14 (Kaysville, UT, USA). A difference with *p* ≤0.05 (*i.e.*, 5%, level of significance) was considered to be statistically significant.

## 4. Results and Discussion

In the present work, a natural polymer *i.e.*, citrus pectin was used to prepare the nanoparticles (CPNs and E-CPNs) bearing 5-FU. For rendering protection against the hostile gastric environment, Eudragit S100 was used to coat the CPNs meant for targeting colorectal cancer. As a polyanion natural polymer, pectin shows ability to crosslink in the presence of Ca^2+^ ions. CPNs were prepared using the method reported by Yu *et al.* (2009) [20] and relatively low concentration of Ca^2+^ was used to avoid complete gelation and formation of bulk gels. The alkaline system (pectin chains remained in stretched conformation) was dialyzed in water to bring the pH to neutral. Nanoparticles were spherical in shape with nanometric size and prominent integrity as shown in Figure 1. Average particle size of CPNs was 174.65 ± 5.32 nm while E-CPNs were of 218.12 ± 10.25 nm in size (Table 1). The zeta potential of the formulations can make a prediction about the stability of colloid dispersions and usually a high zeta potential (>|30| mV) can provide an electric repulsion to avoid aggregation of particles. The zeta potential of CPNs was found to be negative *i.e.*,−18.4 ± 0.4 mV due to partial saturation of free galacturonic acid groups of pectin by Ca^2+^ ions whereas E-CPNs showed −27.5 ± 0.8 mV zeta potential attributed to the presence of free acrylic acid groups on the surface. The percent entrapment efficiency of CPNs was determined after lysing the nanoparticles in PBS (pH 7.0) containing pectinase (120 FDU/mL). It was found to be 38.75% ± 0.74% and 35.15% ± 0.52% for CPNs and E-CPNs (Table 1).

*In vitro* drug release from the nanoparticles was assessed in simulated gastrointestinal fluid mediums of different pH according to the Souder and Ellenbogen extraction technique [23] using a modified USP dissolution test apparatus (Apparatus type 2). The *in vitro* release profile of E-CPNs (Figure 2) clearly exhibited negligible leaching of the drug at pH 4.5 as compared to more than 20% drug release from CPNs. 

In the case of CPNs, the release rate increased with increase in pH. At pH 1.2, the carboxyl groups of pectin are protonated, and the nanoparticles shrink and aggregate, leading to the slow release rate. As the pH increases, the number of negatively charged carboxylate groups increases and thus, the swelling ratio increases due to the ionization of carboxyl groups leading to repulsion in the pectin chains [32]. As a result, the release rate from nanoparticles increases with the increasing pH values of the release medium. The release data are in accordance with the fact that nanoparticles at SCF (pH 7.0) could have a high swelling ratio which leads to enhancement in permeability of the pectin matrix. The drug release was also favored by degradation of pectin due to pectinase (120 FDU/mL) which is found to be responsible for digestion of pectin *in vivo*. In the case of E-CPNs, there was no significant drug release up to 4 h. Drug release began only after 4 h in simulated intestinal fluid (pH 6.8). This can be explained by the fact that the Eudragit S100 polymer contains carboxyl groups that ionize when the pH switches from acidic to alkaline. As the ionization takes place, the integrity of the coat is disturbed and the drug starts leaching from the nanoparticles [33]. In the case of CPNs, drug release was found to be 74.38% ± 3.2%and 98.32% ± 4.20% at the end of 8 and 24 h, respectively while E-CPNs showed drug release of 57.64% ± 2.1% and 79.24% ± 3.15% at the end of 8 and 24 h, respectively (Figure 2).

The *in vitro* drug release study of CPNs was also carried out in PBS (pH 7.0) with and without rat caecal content at 24 h. The results showed ~60% release of drug from CPNs opposed to ~20% from E-CPNs in PBS (pH 7.0) without rat caecal content. While in the medium containing 2% rat caecal content, there were ~92% and ~60% drug release for CPNs and E-CPNs, respectively. The results showed improved drug release as compared to control (PBS pH 7.0) attributed to the various anaerobic bacteria present in caecum responsible for the digestion/degradation of the pectin facilitating release of drug from the nanoparticles (Figure 3) [34,35]. The controlled release profile of 5-FU from E-CPNs in our study corroborated well with those reported previously in addition to negligible release of 5-FU at lower pH (1–2) as compared to previously reported studies [36,37].

The SRB assay is based on the ability of the protein dye Sulforhodamine B to bind electrostatically in a pH dependent manner on protein basic amino acid residues of trichloroacetic acid-fixed cells. Under mild acidic conditions it binds, while under mild basic conditions it can be extracted from cells and solubilized for measurement [38]. Table 2 summarized the LC_50_, TGI and GI_50_ values of various formulations. Nanoparticles (CPNs and E-CPNs) showed significant cytotoxic effect and reduction in cell growth compared to free drug solution after 48 h incubation period in HT-29 cancer cell lines (*p* < 0.05). The amount of uncoated nanoparticles (CPNs) required to achieve 50% of growth inhibition (GI_50_) was 1.5 fold lower than that of drug solution. This enhanced cytotoxic potential may be accounted for by the improved cellular internalization via galectin-3 receptors that over express in HT-29 colorectal cancer cell line [39]. Additionally, it could be due to anti-proliferative activity of citrus pectin by activation of caspase-3 pathway [40,41]. In the case of coated nanoparticles (E-CPNs), the cytotoxicity was significantly reduced as compared to CPNs which might be due to a number of reasons: (1) Firstly, Eudragit S100 having no self cytotoxicity potential on cell lines like HT-29, VK2, HeLa as reported by Yoo *et al.* (2011) [42]; (2) Secondly, the enteric coating unable to dissolve at the acidic pH of the medium used in the cell cytotoxicity study. However, little cytotoxicity of E-CPNs could be attributed to nominal leaching of drug over a period of 48 h of incubation. These results demonstrated that the incorporation of the drug in CPNs significantly enhanced the cytotoxic potential of the drug. Moreover, enteric coating successfully protected the nanoparticles in an acidic environment which was useful to establish *in vitro–in vivo* correlation. The growth curves exhibited control of growth in a concentration dependent manner in all formulations but CPNs showed superiority over the 5-FU control (Figure 4).

**Table 2:** Cytotoxicity potentials (SRB assay) of nanoparticles in HT-29 cancer cell lines.

Formulation	LC_50_ (μg/mL)	TGI (μg/mL)	GI_50_ (μg/mL)
5-FU solution	56.7 ± 4.2	33.7 ± 2.4	9.3 ± 0.7
CPNs	36.4 ± 3.2	25.8 ± 1.8	6.5 ± 0.4
E-CPNs	94.2 ± 3.8	76.4 ± 2.2	18.5 ± 0.5

Values represent Mean ± SD (*n* = 3, *p* < 0.05).

*In vivo* studies are usually conducted to evaluate the site specificity of drug release and to obtain relevant pharmacokinetic information of the delivery system. Factors like pH of various biological fluids, enzyme system, and interaction of the dosage form with the biological environment are likely to influence the performance of the dosage form. *In vitro* cytotoxicity assay was carried out with an aim to establish the superiority of the developed nanoparticles, if any, over the 5-FU control. Since, the developed nanoparticles were intended for use against colorectal cancer, HT-29 human colon cancer cell line was chosen for the assay. The *in vivo* biological performance *i.e.*, the GI distribution of any drug loaded pectin nanoparticles has not been previously reported. Although some studies relating to the biodistribution of pectin microspheres and beads in mice have been reported [36,43,44]. CPNs andE-CPNs were evaluated for *in vivo* performance in albino rats (Wistar strain) to speculate the evidence on their clinical applications. The percent drug recovered in different parts of the GIT is graphically presented in Figure 5.

The maximum recovery of FU (*i.e.*, 59.80% ± 4.83%) was observed after 2 h in the stomach following oral administration of 5-FU solution and in subsequent hours; much less drug reached the small intestine, and negligible amount of drug was found in the colon. Less drug release from CPNs in the small intestine and colon might be due to the shrinkage of CPNs in the acidic environment (gastric milieu) leading to a more compact structure and thereby restricting the release of drug. While in the case of coated nanoparticles (E-CPNs), release was more hindered due to the additional enteric coating of Eudragit S100. Nominal recovery of 5-FU from CPNs in the stomach was accounted to erosion of the adhered drug to the surface CPNs and a phenomenal shrinking of CPNs in acidic milieu.

In the small intestine region (with fluids), the maximum amount of drug was recovered after 4 h of oral administration of different formulations. The amounts of drug recovered after 4 h were 12.29% ± 0.98%, 42.10% ± 2.95%, 4.24% ± 0.35% for 5-FU solution, CPNs and E-CPNs, respectively (Figure 5). This might be attributed to the different release profiles of the formulations. In the case of 5-FU solution, the maximum amount of drug might have been absorbed through the stomach wall to systemic circulation thereby a lesser amount would reach the small intestine. However, in the case of CPNs, a relatively greater amount was recovered from the small intestine which might be due to the following reasons. Firstly, restricted release of drug in acidic medium as described earlier thereby resulting in a greater amount reaching the small intestine. Secondly, the presence of abundant microflora (~10^2^–10^7^ counts/g) along the whole length of the small intestine might have facilitated the drug release by enzymatic degradation of pectin. E-CPNs, being guarded by Eudragit S100 cover, showed the least drug recovery from the anterior part of the small intestine as compared to other formulations but it probably exhibited release in the ileo-caecal region *i.e.*, the posterior part of the small intestine which provided a favorable pH to uncover the nanoparticles from the coating. In subsequent hours, a lesser amount of drug was found in the small intestine which might be due to the drug absorption and transit through the intestine [36].

After oral administration of various formulations, the biodistribution results for the colon that included the caecal contents, caecum tissue, colon contents, and colon tissue are demonstrated in Figure 5. The maximum drug recovery was observed in the case of E-CPNs after 8 h showing the peak level of 5-FU *i.e.*, 92.90% ± 4.65% as compared to 15.09% ± 1.88% for 5-FU solution and 62.47% ± 3.65% for CPNs. The results confirmed that the least amount of drug was lost in the upper GIT from E-CPNs attributed to the protective coat. Moreover, enhanced increase in 5-FU concentration in the colon in the case of E-CPNs was noticed because of facilitated degradation by the microflora in the colonic region [45]. There was enhanced accumulation of 5-FU at the colon *i.e.*, 200.48 μg 5-FU/g rat colon which was better than in previously reported studies [46].

In order to evaluate drug absorption, plasma drug profile was established in terms of the plasma concentration *vs.* time profiles of 5-FU after oral administration of various formulations to the rats (7.14 mg/kg) and is depicted in Figure 6. After oral administration of the 5-FU solution, the drug was detected rapidly in plasma in the initial hours, attributed to the higher permeability coefficient of 5-FU in the upper GIT. Thereafter, the drug-plasma concentration decreased quickly to undetectable levels after 8 h. These results were in accordance with the reports published by Zinutti *et al.* (1998) [47] and Li *et al.* (2008) [30]. In the case of CPNs, delayed and slow absorption was observed as 5-FU might not have been released in the GI tract from where the drug is absorbed relatively at a faster rate. However, it was released in the small intestine from where the drug showed slow absorption due to low permeability [45]. In the case of E-CPNs, the maximum 5-FU level was reached after 12 h of oral administration and then gradually decreased over the next 12 h, which indicated the prolonged residence time of the released drug in the colon with slow leaching of the drug to systemic circulation due to low permeability and compromised surface area [48,49]. 

The colon, acts like a homogeneous reservoir to elicit slow and constant drug input which is beneficial to the cancer therapy in the case of drugs with short plasma half-lives. The steady low plasma drug concentrations in the case of E-CPNs can provide not only a safety benefit by reducing the magnitude of peak plasma drug levels [50] but also result in sustained drug exposure of cancer cells [51].

## 5. Conclusions

The conventional chemotherapeutic approach has not been found to be very effective in colorectal cancer as the drug molecule does not reach the target site in effective concentrations. Pectin could be a promising carrier material in colon-specific drug delivery systems as data of studies conducted showed that Eudragit S100 coated CPNs (E-CPNs) were safe and effective *in vitro* and *in vivo* studies to deliver 5-FU for the effective treatment of colorectal cancer. Citrus pectin possessing multiple features also acts as a ligand for galectin-3 receptors that are over expressed on colorectal cancer cells and was found to enhance the targeting potential of E-CPNs to cancer cells.

## Supplementary Information

Supplementary materials can be accessed at: http://www.mdpi.com/1996-1944/8/3/0832/s1.
