# Supplementary material for: Eudragit S100 Coated Citrus Pectin Nanoparticles for Colon Targeting of 5-Fluorouracil
Source: Materials (Basel). 2015 Feb 27;8(3):832–49. doi: 10.3390/ma8030832 (PMC5455456; doi:10.3390/ma8030832)
Supplement: Supplementary file 1 [file materials-08-00832-s001.pdf]

Supplementary Information

Coating of Eudragit S 100 on Citrus Pectin Nanoparticles

The coating of Eudragit S 100 on Citrus Pectin Nanoparticles (CPNs) was done by simple solvent evaporation method as reported by Maestrelli *et al.* (2008). The enteric coating solution was prepared by dissolving Eudragit S 100 in acetone at 12% w/v. Coating was obtained by dispersing 50 mg of nanoparticles in coating solution with a definite Core: Coat ratio followed by solvent evaporation in Rotary Evaporator (Super Fit, Ambala, India). The process was repeated until the desired amount of coating was achieved. Samples of coated nanoparticles were then dried and weighed.

Optimization of Core: Coat ratio

The Core: Coat ratio was optimized in terms of particle size, shape and size distribution. The coating was done with Core: Coat ratio 1:5, 1:10 and 1:15 (w/w). The results are given in the Table below.

Table S1. Optimization of Core: Coat ratio.

Formulation code	Core: Coat ratio	Average size (nm)	Shape	PDI
CNP1	1:5	184.67 ± 9.36	Uncoated particles seen	0.129
CNP2	1:10	218.12 ± 10.25	Spherical with uniform	0.117
CNP3	1:15	245.94 ± 11.58	Aggregates	0.157

Values represent Mean ± SD, n = 5.
